# Early Implications for Solid Organ Transplantation With the Use of Artificial Intelligence From a Bibliometric Perspective

**DOI:** 10.1016/j.mcpdig.2026.100340

**Published:** 2026-01-30

**Authors:** Aliza Naomi Márquez Cabral, Carlos Alejandro Martínez-Zamora, Oscar Abraham José Padilla Solís, Ángel Lee, Alejandro Rossano García

**Affiliations:** a Social Service, Grupo Médico Rossano, Universidad del Valle de México, Ciudad de México, México; b Hospital Ángeles Acoxpa, Ciudad de México, México; c Saint Luke, School of Medicine, Ciudad de México, México; d Universidad de Guanajuato, Guanajuato, México; e Hospital Ángeles del Pedregal, Ciudad de México, México; f Transplant and Hepatopancreatobiliar Surgeon, Grupo Médico Rossano, Hospital Ángeles Pedregal, Ciudad de México, México

## Abstract

Artificial intelligence (AI) is increasingly transforming health care, particularly in solid organ transplantation, where it addresses complex challenges such as organ allocation, graft rejection prediction, and immunosuppressive management. This bibliometric analysis evaluated the scientific impact and evolution of AI applications in kidney, liver, heart, and lung transplantation. A comprehensive search across PubMed, Scopus, and Web of Science identified 2384 publications from 1989 to 2025, of which 815 met inclusion criteria after double-blind screening with Rayyan AI. Coauthorship, keyword co-occurrence, and collaboration networks were analyzed using VOSviewer and Bibliometrix. The United States led in publications, citations, and collaboration strength, with Mayo Clinic emerging as the most productive institution, followed by China. Machine learning, expert systems, and deep learning were the most frequently applied AI techniques, whereas kidney and liver transplantation were the most extensively studied. Thematic clusters included rejection prediction, patient survival, organ allocation, postoperative monitoring, and immunosuppression personalization. Artificial intelligence–driven models integrate clinical, immunological, histological, and imaging data to enhance predictive accuracy, support clinical decision making, and improve graft and patient outcomes. Although many of these models remain under validation, early findings indicate strong potential to optimize patient care and surgical outcomes. This study highlights global research trends and emphasizes the need for interdisciplinary collaboration to develop context-specific AI tools. Moreover, promoting bibliometric literacy among health care professionals may strengthen evidence-based research and accelerate the responsible integration of AI into transplant medicine.

**Article Highlights**

• Global bibliometric overview of artificial intelligence applications in solid organ transplantation.
• The United States and China identified as the leading contributors to scientific production in this field.
• Machine learning, expert systems, and deep learning emerge as the predominant artificial intelligence methodologies.
• Major clinical applications include graft survival, patient prognosis, and optimization of organ allocation.
• Future directions emphasize interdisciplinary collaboration and region-specific innovation.

Solid organ transplantation (SOT) is a fundamental aspect of contemporary medicine, providing life-saving interventions for patients with end-stage organ failure.^1^ Organ transplantation plays a pivotal role in global medicine and has emerged as a crucial therapeutic approach for preserving and extending life across diverse clinical scenarios. According to the Global Observatory on Donation and Transplantation, a total of 172,397 solid organ transplants were performed worldwide in 2023.^2^ The kidney was the most frequently transplanted organ in 2023, with a total of 111,135 procedures performed. It was followed up by the liver with 41,099 transplants, the heart with 10,121, and finally the lungs with 7811 transplants.^2^ Data from 2016, as reported by the US Organ Procurement and Transplant Network and the Scientific Registry of Transplant Recipients, indicate that the 5-year graft survival rates have reached 80% for renal transplants and 70% for liver transplants.^3^

One of the first artificial intelligence (AI) programs to evaluate chemical data in medicine was The Dendral System, created in 1964.^4^ This advancement paved the way for medical data analysis. Stanford University’s 1970s AI expert system MYCIN was a pioneer. It helps doctors diagnose and treat bacterial infections.^5^ These early breakthroughs had minimal impact on clinical practice because neither national nor international agencies regulated their use. The Food and Drug Administration–approved IDx-DR, the first AI program for primary care diabetic retinopathy screening, in April 2018.^6^ The use of AI in diagnosis, therapy, population health management, monitoring, administration, and regulation changed with this milestone.^7^ Machine learning (ML) has been a major aspect of AI since Arthur Samuel proposed the term in 1959. Machine learning is an AI area that lets computers learn from experience to improve their performance. Without explicit programming for every circumstance, ML systems examine data, detect trends, and change their behavior, improving over time through data-driven learning.^8^

The use of AI and ML into organ retrieval and transplantation operations signifies a viable strategy to tackle these difficulties and improve the efficiency and efficacy of transplant procedures.^9^ Progress in robotics and AI has considerably enhanced the results and success rates of transplant surgery. Transplant surgeons have surmounted numerous challenges by amalgamating the precision afforded by robotic technology with the analytical capabilities of AI algorithms, ushering in an era of enhanced efficacy and accessibility in organ transplantation.^10^ The utilization of AI in donor-recipient matching enables a more sophisticated and data-informed methodology for organ distribution. Conventional matching criteria reliant on blood type and tissue compatibility are now enhanced by AI algorithms, which can analyze extensive patient data in real time. This skill increases the likelihood of transplant success and decreases the duration during which the patient is off the waiting lists, thereby alleviating the dangers associated with prolonged organ failure.^11^^,^^12^ Furthermore, the influence of AI transcends preoperative stages, affecting surgical planning and intraoperative decision making, such as advanced image processing techniques provide accurate organ segmentation and anatomical mapping from medical imaging data, enhancing tailored surgical methods and reducing intraoperative complications.^13^

From a methodological standpoint, these early and contemporary applications of AI in medicine belong to a broader family of medical diagnostic decision-support systems that combine clinical algorithms, probabilistic models, and expert rule–based reasoning operating on relatively small, highly structured data sets. Historical work in biomedical informatics showed not only that such systems could augment human diagnosticians in well-defined domains but also that their performance and scalability were constrained by the rigidity of their underlying rule sets, the need for continuous expert-driven maintenance of knowledge bases, and the challenges of evaluating diagnostic tools whose behavior depends on both the system and the human user.

Over the past decades, these decision-support paradigms have progressively evolved. Early rule-based and Bayesian systems working on structured variables were followed by ML models and, more recently, deep-learning architectures such as convolutional neural networks capable of automatically extracting features from radiologic and histopathologic images in transplantation. Building on this trajectory, a third analytic phase is now emerging in which large language models (LLMs) and advanced natural language processing (NLP) techniques can exploit unstructured electronic health record narratives, pathology and radiology reports, and patient–clinician communication. In this context, our bibliometric analysis (BA) captures a field in transition: supervised ML approaches on tabular clinical data still predominate in SOT, with deep-learning and LLM-based applications representing a growing but still comparatively small fraction of the literature.

Artificial intelligence improves organ rejection detection, especially biopsy slide analysis.^14^ For kidney transplant rejection, Banff automation uses automatic histological categorization. For a thorough Banff evaluation, clinicians can add histological lesion ratings, related histological findings, non–rejection-related diagnoses, C4d staining, circulating anti-human leukocyte antigen donor-specific antibodies, electron microscopy data, and molecular markers. Automated diagnostics and specified criteria diminish interobserver variability, enhancing rejection diagnosis consistency.^15^ Success was due to treatment and immunosuppressive therapy augmentation.^16^ In the early postoperative period and during stressful events like infections or rejection, immunosuppressive drug dosage must be adjusted to achieve a therapeutic blood level. AI predicts immunosuppressive drug blood levels noninvasively. One of the first AI-based transplant studies predicted cyclosporine blood levels in 101 heart transplant recipients using evolutionary algorithms and symbolic AI.^17^ This example showed how AI could predict immunosuppressive drug blood levels and estimate patient-specific dosages. These treatments aim to improve graft and patient survival and quality of life. After examining posttransplant electrocardiograms, postoperative imaging tests, routine laboratory assessments, worldwide transplant registries, pretransplant patient circumstances, and time since transplantation, AI can predict patient outcomes.^18^

The global BA predominantly examines academic productivity through published scientific literature, encompassing research articles, books, and conference proceedings, to assess research endeavors within a particular domain.^19^ The BA is also valuable for analyzing the intellectual framework of a specific domain within the current literature. The popularity of BA has surged substantialy, accompanied by a rising volume of literature addressing its uses.^20^^,^^21^

## Materials and Methods

**Figure 1:**
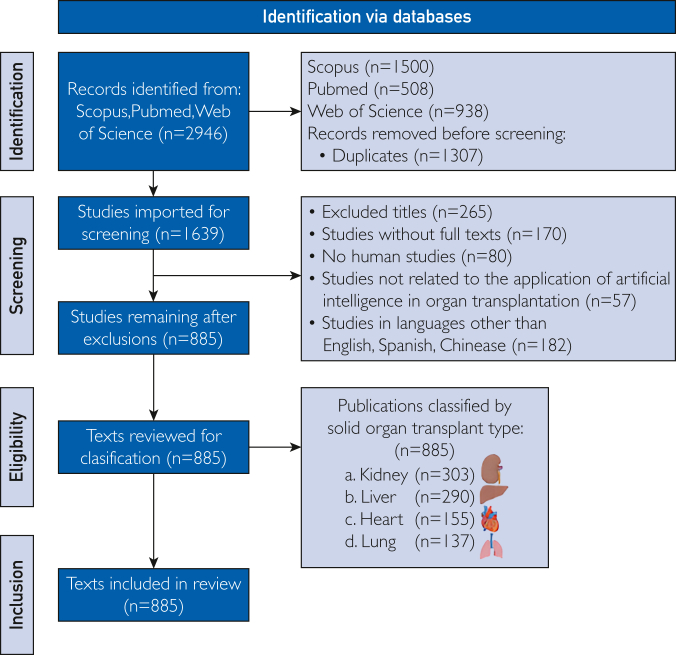
Identification of methodology for search strategy.

A literature search was performed using the Preferred Reporting Items for Systematic Reviewa and Meta-Analyses method (Figure 1) on January 2025. This bibliometric study of the application of AI in SOT used a quantitative method to analyze articles by title indexed in the Scopus, PubMed, and Web of Science databases. The search technique aimed to identify key advances, trends, and research patterns in the convergence of AI and SOT.

The inclusion criteria focused on studies related to the application of AI in vital SOT, specifically the lung, heart, kidney, and liver. The exclusion criteria included all references to cells, tissue, and endocrine-related transplantation. The data collection used the following search terms: TITLE (“Transplant” OR “Transplantation” OR “Graft” OR “Organ transplantation” OR “Donor” OR “Kidney transplantation” OR “Liver transplantation” OR “Lung transplantation” OR “Heart transplantation” OR “Organ procurement” OR “Transplant outcomes” OR “Solid organ transplantation” OR “Donor-recipient” OR “Allograft”)) AND (TITLE (“AI” OR “Artificial Intelligence” OR “Algorithms” OR “Machine Learning” OR “Deep Learning” OR “Digital pathology” OR “Neural Networks” OR “Algorithms classifiers” OR “Big data” OR “Expert systems” OR “Natural Language Processing” OR “Fuzzy logic”)) AND NOT (TITLE (“hematopoietic” OR “stem cell” OR “cell transplantation” OR “myeloma” OR “bone marrow” OR “knee” OR “neural stem cell” OR “blood transfusion” OR “flap” OR “stent” OR “cornea” OR “keratoplasty” OR “apolipoprotein”).

We selected these databases for their comprehensive, diverse, interdisciplinary, and neutral coverage of abstracts and citations, offering a global view of research in medicine and related fields. The BA used quantitative indicators, including annual publication count, distribution by journal, country, institution, author collaboration, and citation impact. All retrieved articles were exported in comma-separated values format, and network visualization tools were applied to explore keyword co-occurrence, author collaboration, thematic evolution, and yearly publication trends (1983-2025). Lists from the 3 databases were independently downloaded, merged, and screened in Rayyan by 2 authors (A.N.M.C. and C.A.M.-Z.) using a double-blind process to remove duplicates and exclude studies not meeting inclusion criteria; disagreements were resolved by a third author (A.R.G.). Each article was classified into one of 18 thematic subcategories, including pretransplant evaluation, organ matching/allocation, donation, organ procurement, immunobiology, immunosuppression, rejection, graft and patient survival, complications, infections, biopsies, prognosis, waiting time, posttransplant monitoring, challenges, applications, limitations, and bibliometrics.

The databases reported the top authors and most cited publications, which our BA monitored. The most cited article were ranked in descending order, and prolific authors were scored by h-index and total publications. The journals with the most and least publications on the topic were identified. The most relevant AI-SOT journals by number of linked publications. *Transplantation* leads with 23 publications, followed by *Journal of Clinical Medicine* and *American Journal of Transplantation. Study in Health Technology and Informatics* is another relevant journal.

**Figure 2:**
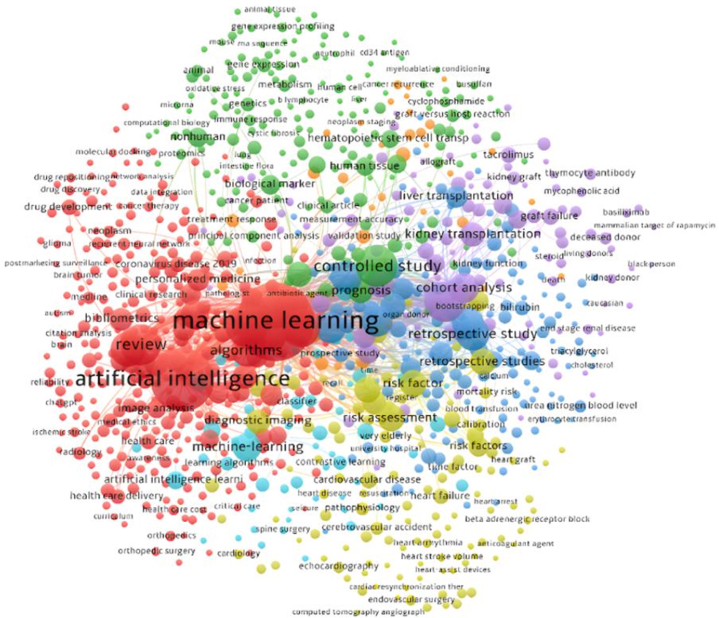
Co-occurrence of keywords (MeSH terms) using Bibliometrix package.

Through the Biblioshiny interface, Bibliometrix 4.0.1 generated co-word analysis, concordance, and cooperation network data. VOSviewer 1.6.20 (Figure 2) was also used. The platform helped us identify the most common phrases used by writers and found a growing prevalence of ML (red cluster) and (blue cluster) with liver transplantation (LT). Kidney (purple cluster) and liver transplants dominate the solid organ market and have the highest transplant rates.

## Results

### Annual and Country Production Analysis

Research on AI in SOT gained momentum around 2010, with a marked surge from government-affiliated institutions after 2014. These institutions not only produced a higher volume of publications but also achieved greater citation impact, indicating both quantitative and qualitative contributions. Early activity (1989-2009) showed modest, steady growth across countries, but from 2015 onward, output accelerated notably, reflecting AI’s increasing integration into clinical and surgical transplantation. The leading role of government institutions highlights their influence in advancing this field. The apparent decline in 2025 output is due to the January data cutoff, not an actual reduction in research activity.

**Figure 3:**
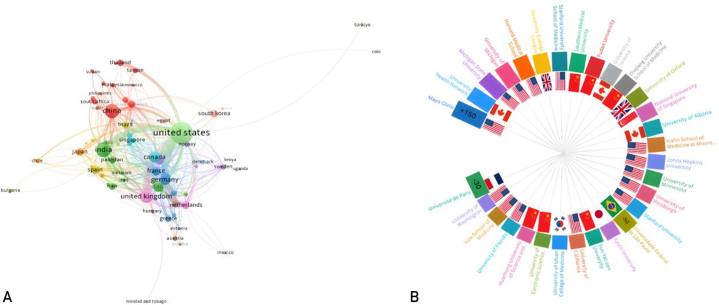
(A) Map depicting international collaboration among countries in research on artificial intelligence applied to critical solid organ transplantation. (B) Radio representation of the 30 most relevant affiliations in scientific production on artificial intelligence and solid organ transplantation.

We want to know how countries/regions having AI in SOT research are related. Figure 3A shows a VOSviewer-created map representing international collaboration on AI-applied critical SOT research. Each node represents a country and its size indicates article volume. Lines connecting nodes represent coauthorship ties, thickening with participation. Globally, 68 countries published articles on AI in crucial SOT. United States leads with 42.69% (n=348) publications and collaborations, followed by China 9.69% (n=79), Germany 6.13% (n=50), the United Kingdom 7.11% (n=58), and Canada 9.20% (n=75). In Latin America, scientific output on this area was far lower than elsewhere. Brazil has published 2.33% (n=19) of this literature, followed by Uruguay with 0.12% (1 article), Chile with 0.49% (4 articles), and Argentina with 0.73% (6 articles). Figure 3A demonstrates regional collaboration clusters with large European networks and strategic links to North America, Asia, and Oceania. This map highlights the field’s global and multidisciplinary nature and specific countries’ crucial roles in information development and dissemination.

The US–China and US–UK connections are the strongest among the high-producing nations, indicating tight cooperation. Strong cooperation exists between China, Asian nations (such as Thailand and South Korea), and United States, indicating that China’s influence in this region is also growing. Regarding the Latin America (LA) region, we observe that the contributing countries—Uruguay, Chile, Argentina, and Brazil—are interconnected, indicating a modest level of collaboration among them. However, a notable relationship is evident between Brazil, the leading producer in LA, and the European continent through its collaboration with Portugal.

Total link strength analysis revealed the United States as the primary global hub for scientific collaboration in AI and SOT, with a total link strength of 7157—far surpassing any other country—driven largely by institutions such as Mayo Clinic. China ranked second (1218) but showed lower global connectivity. Canada (677), Spain (745), and France (511) displayed moderate, regionally focused collaboration, whereas Germany (489), Italy (326), and the United Kingdom (287) reflected intermediate engagement. South Korea (300) and Australia (142) indicated growing participation, whereas countries such as Brazil, Romania, and Switzerland remained more isolated. These disparities highlight the United States’ central role and the uneven integration of nations into global AI–transplant research networks.

### Institutional Analysis

The BA allows us to quantify across all organizational levels in each country, allowing us to identify the institutions and hospitals most involved in this area of SOT research. Figure 3B shows a radial chart illustrating the top 30 universities/institutions in terms of research output in the area of AI applied to SOT. This graphical style makes it easy to quickly identify the most important institutions and those with the least relative commitment. Mayo Clinic stands out with more than 150 articles, reinforcing its status as the pre-eminent center for knowledge development in the field. Conversely, universities such as the Federal University of São Paulo and the University of Paris occupy the lowest positions in the ranking, with less than 50 and 30 publications, respectively.

### Data Analysis

During our analysis of the selected articles, we observed that the most frequently mentioned organ was the kidney in 43.31% (n=353). This finding is consistent with the fact that the kidney is the most commonly transplanted organ worldwide, and therefore, it is assumed to be the most extensively studied. The next most frequently mentioned organ was the liver 32.76% (n=267), followed by the heart 14.35% (n=117) and the lungs 7.23% (n=59). This sequence also mirrors the global frequency of SOT. Additionally, some articles referred collectively to the group of organs analyzed in this study; these were categorized under the label solid organ 2.45% (n=20). A few articles talked about more than 1 organ, so the amount of articles does not correspond with our total.

After examining the most often published subjects through the defined subcategories, we noticed that the primary focus of AI in transplantation is the assessment of graft survival and functionality 15.95% (n=130). Next is 13% patient survival (n=106). Both subjects are related and often discussed simultaneously. Third rank goes to challenges, applications, and limitations (9.69% [n=79]), indicating interest in AI in SOT. Then, prognosis (9.45% [n=77]) and pretransplant assessment (7.23% [n=59]) investigations follow. Other relevant subjects include complications (6.74% [n=55]), organ matching and allocation (6.5% [n=53]), immunosuppressive drug management (6.01% [n=49]), and biopsy analysis (5.76% [n=47]). Graft rejection was (4.66% [n=38]), posttransplant surveillance (3.92% [n=32]), and infections (3.06% [n=25]). Additional categories include various subjects (2.33% [n=19]), solid organ donation (2.33% [n=19]), waiting list times (2.08% [n=17]), transplant immunobiology (1.22% [n=10]), and organ procurement (0.61% [n=5]). In conclusion, BA was 0.36% (n=3).

During the BA, publications were categorized based on the subtype of AI implemented. Machine learning was identified as the predominant methodology, serving as the primary analytical tool (47.23% [n=385]). The general term “artificial intelligence” represents 21.22% (n=173), often encompassing the broader conceptual framework and its subfields as applied to strategy development in SOT. Expert system algorithms were reported in 12.7% (n=104), followed by deep learning (9.69% [n=79]), neural networks (5.15% [n=42]), big data analytics (2.20% [n=18]), digital pathology (0.98% [n=8]), and fuzzy logic systems (0.98% [n=8]). These findings underscore the dominant role of ML in AI-driven applications within the SOT field.

**Figure 4:**
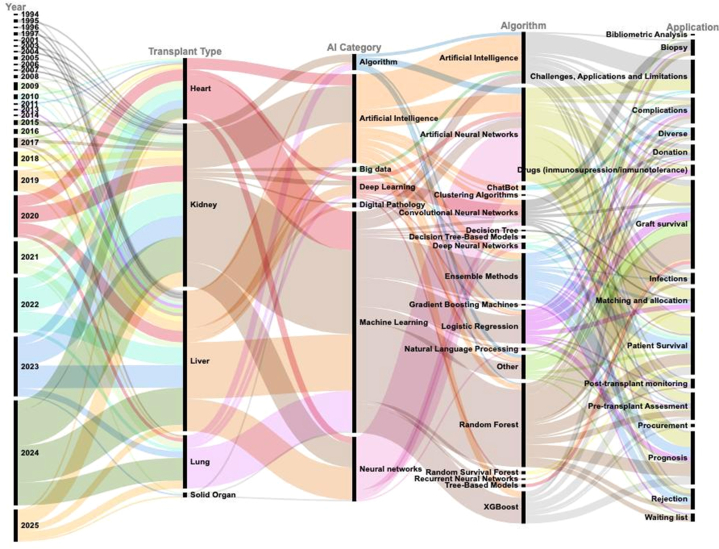
Sankey diagram illustrating the relationships between transplant type, artificial intelligence subtype, algorithm, and clinical application across 225 key articles based on most cited articles, under Bibliometrix criteria, with articles until February 2025.

To examine AI application patterns in SOT, we analyzed 225 key articles from our bibliometric review. A Sankey diagram mapped connections between publication year, transplant type, AI subtype, algorithm, and clinical application (Figure 4). Kidney was the most studied organ, followed by the liver, heart, lung, and general SOT. Machine learning predominated, with 2024 as the peak year. Artificial neural networks, random forest, logistic regression, and ensemble methods were the most used algorithms, mainly applied to predicting graft survival, patient survival, prognosis, and pretransplant assessment. These findings highlight the increasing precision and clinical alignment of AI tools in transplantation.

We noticed that numerous articles in our review were rarely mentioned, perhaps due to the topic’s recent peak or early access status. The 2020 *Hepatology* article “Applying machine learning in liver disease and transplantation: a comprehensive review” by US-based authors was the most referenced. This 148-citation article examines machine learning’s predictive utility in hepatology and LT, including imaging analysis, survival prediction, and posttransplant prognosis. Table 1^4^^,^^22^, ^23^, ^24^, ^25^, ^26^, ^27^, ^28^, ^29^, ^30^, ^31^, ^32^, ^33^, ^34^, ^35^, ^36^, ^37^, ^38^, ^39^, ^40^ lists our review’s 20 most cited publications. Seven of the top 20 most cited articles were written by US researchers, confirming that the United States is the most cited nation. Despite being second in publishing production, China has only 2 articles in the top 20 most referenced list and ranks sixth in nation citations. Italy ranks second in top-cited articles with 3, reflecting its productivity. Although Spain has fewer publications than China or the United Kingdom, it is second in citation count and has 2 top-20 papers, one of which rates second. Romania is represented in the top 20 cited systematic review publications despite its low publishing output.

**Table:** The 20 Most Cited Articles in the Field

Title	Authors	Country/year	Organ	Journal	Citations
Applying machine learning in liver disease and transplantation: A comprehensive review	Spann A	US/2020		*Hepatology*	148
Use of artificial intelligence as an innovative donor-recipient matching model for liver transplantation: Results from a multicenter Spanish study	Briceño J	ES/2014		*Journal of Hepatology*	106
Fuzzy logic based clinical decision support system for the evaluation of renal function in post-transplant patients	Improta G	IT/2020		*Journal of Evaluation in Clinical Practice*	89
Artificial intelligence, machine learning, and deep learning in liver transplantation	Bhat M	US/2023		*Journal of Hepatology*	65
An explainable supervised machine learning predictor of acute kidney injury after adult deceased donor liver transplantation	Zhag Y	CN/2021		*Journal of Translational Medicine*	52
Promises of big data and artificial intelligence in nephrology and transplantation	Thongprayoon C	US/2020		*Journal of Clinical Medicine*	49
Novel model to predict HCC recurrence after liver transplantation obtained using deep learning: A multicenter study	Nam JY	KR/2020		*Cancers*	48
Using artificial intelligence for predicting survival of individual grafts in liver transplantation: A systematic review	Wingfield LR	GB/2020		*Liver Transplantation*	46
An imageomics and multi-network based deep learning model for risk assessment of liver transplantation for hepatocellular cancer	He T	US/2021		*Computerized Medical Imaging and Graphics*	43
Using artificial intelligence resources in dialysis and kidney transplant patients: A literature review	Burlacu A	RO/2020		*BioMed Research International*	41
Use of artificial intelligence as an innovative method for liver graft macrosteatosis assessment	Cosaretti M	IT/2020		*Liver Transplantation*	37
Artificial intelligence and algorithmic computational pathology: An introduction with renal allograft examples	Farris AB	US/2021		*Histopathology*	34
Application of artificial intelligence techniques to predict survival in kidney transplantation: A review	Diez-Sanmartin C	ES/2020		*Journal of Clinical Medicine*	32
Machine learning in liver transplantation: A tool for some unsolved questions?	Farrarese A	IT/2021		*Transplant International*	26
Application of machine learning models for predicting acute kidney injury following donation after cardiac death liver transplantation	He ZL	CN/2021		*Hepatobiliary and Pancreatic Diseases International*	25
Technology-enabled care and artificial intelligence in kidney transplantation	Scwantes IR	US/2021		*Current Transplantation Reports*	25
Artificial intelligence to identify harmful alcohol use after early liver transplant for alcohol-associated hepatitis	Lee BP	US/2022		*American Journal of Transplantation*	24
Artificial intelligence: Present and future potential for solid organ transplantation	Peloso A	CH/2022	SOT	*Transplant International*	24
Machine learning and artificial intelligence in cardiac transplantation: A systematic review	Naruka V	GB/2022		*Artificial Organs*	24
Artificial intelligence improves estimation of tacrolimus area under the concentration over time curve in renal transplant recipients	Niel O	FR/2018		*Transplant International*	23

SOT, solid organ transplantation.

### Analysis of Authorship

A total of 5150 authors have contributed to AI in organ transplantation. The most cited in our data set was Peter A. Noseworthy (337 citations, 7 publications, h-index = 53), followed by Zachi I. Attia (331 citations, h-index = 44) and Francisco López-Jiménez (329 citations, h-index = 84). Paul A. Friedman, with 328 citations, stands out for his prolific output (838 publications, >28,000 citations) and contributions to AI-assisted diagnostics. Other notable contributors include Wisit Cheungpasitporn (280 citations, h-index = 38), Charat Thongprayoon (275 citations, h-index = 45), Noppadol Leeaphorn (260 citations), and Michael Cooper (240 citations, h-index = 105). High citation density and collaborative networks highlight their growing influence, particularly in nephrology and transplantation.

The temporal distribution of author productivity over 3 consecutive 5-year periods reveals key patterns in the evolution of research on AI in SOT. Between 2015 and 2019, scientific output was relatively limited, with a marked increase around 2020. During this stage, Wisit Cheungpasitporn emerged as a leading contributor. In the subsequent 2016-2020 period, Peter A. Noseworthy gained prominence as one of the most influential authors in the field, and international collaboration began to expand, including co-authorships with researchers such as Alexandre Loupy (France) and other non-US collaborators. From 2020 to 2025, productivity remained comparable with the preceding period, with a peak in 2024 and sustained contributions from the previously identified leading authors.

Coauthorship analysis identifies a dominant Mayo Clinic cluster in cardiac transplantation, led by Peter A. Noseworthy, Paul A. Friedman, Patricia A. Pellikka, and Erika Douglass, with Zachi I. Attia contributing AI and signal processing expertise. Another Mayo group, led by Wisit Cheungpasitporn with Pooja Budhiraja and Jing Miao, focuses on AI in kidney transplantation (KT). Mamatha Bhat (University of Toronto) leads a LT cluster with US collaborators, whereas Alexandre Loupy, Marta Crespo, and Tanjala S. Purnell address epidemiological aspects of AI in SOT. Italian research, led by Patrizia Burra, and smaller European groups highlight regional collaboration. Strong US interconnectivity and crosscluster links underscore active international cooperation in advancing AI for transplantation.

## Discussion

The integration of AI into SOT is transforming modern medicine, enhancing decision making, patient outcomes, and long-term graft survival.^41^ Our BA shows a growing global adoption of AI, with ML emerging as the leading driver of innovation. The literature highlights its use in KT, one of the most in-demand solid organs.^42^ Early AI in transplantation relied on rule-based systems, fuzzy logic, and expert tools.^41^^,^^43^ Machine learning now dominates, representing 385 publications, and excels in analyzing complex, high-dimensional data to model outcomes with greater accuracy than traditional statistics. It has shown strong performance in predicting graft survival, acute rejection, drug levels, and complications, enabling early risk detection, personalized therapy, and better clinical results.^44^

These patterns are consistent with the broader historical evolution of diagnostic decision-support systems in medicine. Classic medical diagnostic decision-support systems architectures based on logical rules, Bayesian reasoning, and heuristic matching were originally conceived for narrow, well-structured problems and required painstaking construction and maintenance of medical knowledge bases.^45^ Subsequent generations of systems increasingly leveraged data-driven models, with supervised ML algorithms becoming the dominant analytic approach in our bibliometric sample, and deep-learning methods progressively expanding into imaging and digital pathology. Despite growing interest in LLMs and other advanced NLP techniques, our findings indicate that LLM-based applications in SOT remain at an early stage compared with traditional ML, suggesting that the field is still in a transition phase from predominantly structured, model-driven analytics toward more flexible architectures capable of integrating heterogeneous clinical, imaging, and textual data.^46^

Artificial intelligence has also demonstrated strong potential in pretransplant evaluation, encompassing donor-recipient matching, organ assessment, and recipient risk stratification.^15^ Current methods guiding these steps are often labor-intensive, subjective, and inconsistent. Artificial intelligence can address these limitations by providing standardized, data-driven models that increase both efficiency and diagnostic precision. For instance, AI-powered imaging analysis improves preoperative planning and graft quality assessment,^15^^,^^47^^,^^48^ whereas ML models optimize immunosuppressive therapy and long-term follow-up.^49^ Beyond ML, other AI subtypes such as expert systems (n=104), deep learning (n=79), and neural networks (n=42) have contributed meaningfully to the field. These technologies have enabled advancements in histopathological analysis, image-guided diagnostics, and complex clinical decision-support tools. Notably, AI systems like the Banff Automation System now assist in automating biopsy interpretation and classification of rejection episodes, reducing interobserver variability and increasing diagnostic reliability.^50^

In evaluating AI tools used in transplantation, it is essential to recognize that each analytic paradigm achieves optimal performance under different data structures. Classical ML models—currently, the most frequently reported in the transplant literature—commonly rely on quantitative metrics such as area under the receiver operating characteristic curve, precision, recall, and the F1-score, which is particularly informative in the presence of substantial class imbalance such as rare rejection or graft-failure events.^50^^,^^51^ Deep-learning models applied to radiology and digital pathology are typically evaluated using Dice similarity coefficients, sensitivity–specificity matrices, and concordance with expert reviewers because these metrics better capture segmentation and pattern-recognition performance in high-dimensional imaging tasks.^52^ However, these models require large, accurately annotated data sets, and their performance declines markedly when ground-truth labels are noisy or inconsistently defined.

In contrast, emerging LLM-based systems that operate on unstructured clinical narratives—such as electronic health record notes, pathology reports, or patient–clinician communication—use evaluation frameworks derived from NLP. These include token-based metrics such as bilingual evaluation understudy and recall-oriented understudy for gisting evaluation, alongside task-specific clinical metrics such as extraction accuracy or F1 scores for variable identification, although formal clinical evaluation of LLMs in transplantation remains limited.^53^ Importantly, LLM reliability is highly sensitive to ambiguously defined or heterogeneous clinical outcomes, which remain common in transplant data sets and can reduce model calibration and confidence.^54^

These differences highlight why, at present, ML and CNN-based models are closer to prime-time deployment in transplantation: they perform robustly when trained on structured, discrete, and well-annotated data sets, whereas confusion and reduced reliability arise when algorithms are applied to ill-defined, weakly labeled, or multimodal data. For bibliometric interpretation, this analytic heterogeneity contextualizes why the field remains dominated by classical ML techniques, with relatively few studies exploring LLM-based applications. As such, bibliometric patterns should be understood as reflecting methodological adoption rather than analytic accuracy, and the intent of each study—whether diagnostic imaging, outcome prediction, allocation modeling, or patient-centered communication—must be considered when interpreting the observed trends.

As bibliometric research provides a macrolevel mapping of scientific activity rather than an evaluation of model performance or predictive accuracy, its purpose is to characterize the structure, trends, and methodological evolution of a field rather than validate AI models.^55^

### Geographic Analysis

Despite these advances, our findings highlight persistent geographic and organ-specific disparities. Kidney transplantation dominates AI-related literature, likely owing to its higher global frequency and availability of structured data sets. In contrast, liver, heart, and lung transplants are comparatively underrepresented. Similarly, regional analysis revealed a concentration of scientific output in high-income countries, particularly the United States and China, with substantialy lower contributions from LA and Africa. This imbalance reflects inequities in access to digital infrastructure, research funding, and technical expertise, underscoring the need for equitable strategies to democratize AI implementation globally.^56^ The authorship analysis also indicates that the United States is the country with the most highly cited authors, primarily owing to their affiliation with Mayo Clinic, which stands out as the most prolific institution in this field.^57^ The United States has a highly developed SOT system that serves as a global benchmark in terms of infrastructure, volume, and outcomes. More than 140 certified transplant centers perform these procedures, with most centers conducting over 10 transplants annually. In 2024, the country performed 27,759 kidneys, 11,458 liver, 4572 heart, and 3340 lung transplants, totaling over 48,000 organ transplants for the first time in its history.^58^ This represents an increase of 3.3% compared with 2023, with substantial growth observed particularly in liver and lung transplantation. The system is supported by 16,988 deceased and 7030 living donors, yielding transplantation rates of approximately 83 kidneys, 34 liver, 13 heart, and 10 lung transplants per million population (pmp).^59^ These figures position the United States among the highest-performing countries globally in organ transplantation.

It is noteworthy that China, despite ranking second in overall article production, does not appear among the top 5 countries with the most cited authors. This discrepancy may suggest that Chinese publications have relatively lower impact or limited dissemination, potentially influenced by national policies or language barriers.^14^^,^^60^ China has a rapidly expanding SOT system, supported by a growing national infrastructure and increasingly stringent regulations. By the end of 2022, 183 medical institutions were authorized to perform organ transplants, and a total of 20,229 transplant operations were conducted that year, including 12,712 kidneys, 6053 liver, 799 lungs, and 710 heart transplants.^61^ Despite this progress, the country continues to face important challenges. The organ donation rate in China remains relatively low at approximately 3.86 donors pmp, far behind countries like Spain and the United States.^62^ Although more than 6 million people have registered as organ donors, the demand far exceeds the supply, with over 140,000 patients currently awaiting transplantation.^63^

The apparent underrepresentation of Chinese scientific output may stem from limited international visibility of certain publications. Political, regulatory, and language barriers, along with restricted access to some databases, can hinder global dissemination and indexing, potentially underestimating China’s bibliometric impact in AI for SOT.

Spain has a highly advanced and globally recognized SOT system that has led the world for more than 3 decades. In 2024, the country performed a total of 6464 organ transplants, including 4047 kidneys, 1344 liver, and 623 lung transplants, marking a 10% increase compared with that in the previous year.^64^ This was made possible by the generosity of 2562 deceased donors, resulting in a donation rate of 52.6 donors pmp, the highest worldwide.^65^ Over half of lung transplants (30% year-over-year increase), alongside heart and liver transplants, were performed using organs from donors after circulatory arrest—an innovation in which Spain is a global leader.^66^ This outstanding performance not only demonstrates the country’s clinical and logistical capacity but also positions Spain as a benchmark for other nations aiming to improve their SOT programs through systematized coordination and ethical organ allocation frameworks. Spain’s sustained excellence over 30 years, supported by legislative, organizational, and technological innovations, positions it as a model for countries seeking to enhance their SOT systems and leverage this leadership in future AI-driven developments in transplant medicine.

France maintains a robust and internationally respected organ transplant program. In 2024, the country performed approximately 6034 SOT, including kidney, liver, heart, and lung procedures, surpassing prepandemic levels and marking a 7.1% increase compared with that of the previous year.^67^ This growth was supported by a donation rate of approximately 25.8 deceased donors pmp, positioning France among the top countries globally for organ donation activity.^67^^,^^68^

Germany maintains a well-organized and technically advanced transplant program, albeit at a more modest scale compared with leading countries in Europe. In 2023, the German Organ Transplantation Foundation reported 965 deceased donors, resulting in approximately 11.4 donors pmp.^69^ That year, a total of 2877 organs were transplanted, including 1488 kidneys, 766 livers, 303 hearts, and 266 lungs, across 45 national transplant centers.^70^ Although Germany ranks lower in donor rates within the Eurotransplant network, the steady increase after a sharp decline in 2022—an 8.1% rise in organ retrieval—demonstrates a resilient recovery and improved efficiency in the donation system.

Latin America remains in the early stages of AI application in SOT, with fewer publications than North America, Europe, or Asia but notable growth potential.^71^^,^^72^ In LT, Uruguay leads the region with an average of 20.1 donors pmp, sustaining high activity.^73^ Chile also reports comparatively high donation and transplantation rates, supported by a consistent national framework; in 2022, the Clinical Hospital of the Catholic University of Chile performed 66 liver transplants, including 19 from living donors.^71^^,^^72^ Argentina operates under a presumed consent law similar to Chile’s, with structured infrastructure facilitating activity.^74^ Brazil performs about two-thirds of all liver transplants in the region, ranking globally among the most active; with 78 centers, it reported 2022 rates of 9.1 deceased donor liver transplantation and 0.8 living donor liver transplantation (LDLT) pmp, indicating room for growth in LDLT.^75^^,^^76^ Mexico, despite having 84 authorized centers, counts only 23 operational, and just 7 performing >10 procedures annually; in 2022, rates were 1.82 deceased donor liver transplantation and 0.15 LDLT pmp.^77^^,^^78^ Progress in Mexico requires improved infrastructure, public education to boost donation, standardized guidelines to reduce system fragmentation, and greater adoption of expanded criteria donors and living transplantation.

### Contemporary Applications of AI in SOT

Artificial intelligence applications are transforming organ transplantation by enhancing donor-recipient matching and allocation through the integration of diverse data sources, including immunological, clinical, demographic, and genetic variables. Predictive models leveraging these data improve graft survival, optimize allocation efficiency, and reduce waitlist times. However, a key challenge remains in mitigating algorithmic bias because training data sets may inadvertently reflect disparities related to race, gender, or socioeconomic status.^46^^,^^79^ Additionally, AI is increasingly enhancing both predictive analytics and surgical precision in transplantation. Robot-assisted KT has emerged as a minimally invasive technique with promising outcomes, supported by AI-driven tools that contribute to preoperative planning, diagnostic accuracy, risk assessment, and intraoperative optimization. Robotic donor nephrectomy, in particular, has demonstrated improved results compared with conventional approaches. As technological advancements continue to accelerate, AI is poised to play a central role in advancing precision medicine and reshaping the future of transplant surgery.^80^

By enabling the development of personalized immunosuppression strategies, these technologies enhance treatment efficacy, reduce the risk of rejection, and improve posttransplant outcomes.^81^ Moreover, ML models have shown considerable promise in assessing drug exposure levels, such as the area under the curve, and in refining dose adjustments through tools like Bayesian dose optimization systems.^82^ These systems integrate large-scale pharmacokinetic data to support clinicians in delivering precise and individualized care. Additionally, mobile applications for drug monitoring have demonstrated the ability to reduce variability in immunosuppressive drug levels, especially in the critical early posttransplant period. Overall, the integration of AI into immunosuppression management represents a paradigm shift, improving therapeutic precision, patient safety, and long-term graft survival in renal transplantation.^82^^,^^83^ Furthermore, AI systems have outperformed transplant physicians in predicting long-term graft failure, regardless of clinical experience, highlighting their potential as diagnostic aids.^84^^,^^85^

The implementation of LLMs such as ChatGPT in patient education has shown marked potential, particularly for individuals with chronic-degenerative diseases. Studies have indicated that ChatGPT can provide medically relevant information in a generally accurate and understandable manner, thereby supporting patient comprehension and engagement.^86^ It also has shown promise in delivering personalized recommendations for transplant recipients.^87^ Chatbots serve as a helpful resource in this environment, enabling conversation through real-time translation and providing culturally relevant terms and responses.^42^ They facilitate the overcoming of language barriers and enable patients to understand their diagnosis, treatment, and medical procedures, regardless of their proficiency in the primary language of the health care system or their varying levels of health literacy.^88^ This is a useful tool for both patients and physicians. Clinicians can educate patients on their proper use, while emphasizing that AI does not replace in-person medical care. Therefore, it is essential to clearly communicate warning signs that require consultation or urgent attention.

Recent research shows that several ML algorithms have been applied in recent studies to improve the prediction of outcomes in LT.^89^ An artificial neural network model was developed that outperformed traditional end-stage liver disease–based models in predicting short-term mortality.^89^^,^^90^ Introduced an optimized prediction of mortality model based on optimal classification trees, offering enhanced predictive accuracy compared with conventional scoring systems. Beyond short-term outcomes, Kanwal et al^91^ explored ML approaches such as extreme gradient boosting and logistic regression with least absolute shrinkage and selection operator regularization to estimate 1-year mortality in patients with cirrhosis. Collectively, these studies underscore the growing utility of ML algorithms in LT, offering clinicians data-driven tools to support personalized prognostic assessments and potentially guide posttransplant care strategies.

Ethical, legal, and methodological challenges—such as model generalizability, data privacy, algorithmic transparency, and bias—remain key barriers to the adoption of AI in clinical transplantation. Explainable AI frameworks offer a pathway to enhance trust, regulation, and safe integration into high-stakes decisions.^92^ Opportunities include developing robust local databases, creating models tailored to LA genetic and socioeconomic contexts, and expanding AI-supported telemedicine for posttransplant care in remote regions.^4^ Strengthening interdisciplinary training and fostering global collaborations could bridge gaps and position the region as a contributor to AI-driven transplantation.^10^ Beyond improving workflows and surgical outcomes, AI enhances patients’ quality of life by supporting recovery and reducing anxiety through continuous health monitoring.^15^ Moreover, AI-powered chatbots and virtual assistants offer immediate emotional support, fostering safety, reducing isolation, and overcoming geographic and temporal barriers—critical for patients facing prolonged, complex treatments such as SOT. Personalized interactions further strengthen patients’ sense of understanding and trust in care.^93^^,^^94^

This, in turn, contributes to greater satisfaction and a heightened sense of control as patients are granted immediate access to relevant resources and real-time explanations of their treatment plans. Continuous monitoring and feedback facilitated by AI, in coordination with qualified health care professionals, allow patients to visualize their progress, thereby boosting their motivation and optimism throughout the recovery process.^95^

## Conclusion

This BA reveals that ML dominates 47.23% of articles in AI applications for SOT during the past decade, according to this BA. Geographically, the United States leads in publishing volume (42.69%) and collaborative strength, whereas LA contributes less than 3%. Mayo Clinic is the most prolific and collaborative, concentrating knowledge in high-resource locations. Moreover, AI has transformative potential across all transplant phases, from preoperative planning to postoperative monitoring, with promising clinical applications showing 90% to 92% histopathological analysis accuracy and root mean square errors below 10% in drug level predictions. Key limitations exist in creating focused AI tools for clinical use and addressing organ-specific inequities, with KT dominating research and heart and lung transplants underrepresented. The analysis emphasizes the necessity for interdisciplinary collaboration, consistent regulations, and algorithmic bias and data privacy ethics. Latin American scholars should generate context-specific evidence and form regional collaboration networks. Safe and equitable AI technology adoption requires rigorous clinical validation, research infrastructure investment, and international cooperation. This study shows how BA can evaluate scientific landscapes and uncover knowledge gaps, making it an accessible way for training future researchers in evidence-based critical thinking as medicine becomes data-driven.

## Potential Competing Interests

The authors report no competing interests.
